# Epigenetic Age Acceleration Is Not Associated with Age-Related Macular Degeneration

**DOI:** 10.3390/ijms222413457

**Published:** 2021-12-15

**Authors:** Neil Saptarshi, Daniel Green, Angela Cree, Andrew Lotery, Luminita Paraoan, Louise F. Porter

**Affiliations:** 1 Department of Eye and Vision Science, Institute of Life Course and Medical Sciences, University of Liverpool, Liverpool L7 8TX, UK; N.saptarshi@liverpool.ac.uk; 2 Department of Musculoskeletal Biology, Institute of Life Course and Medical Sciences, University of Liverpool, Liverpool L7 8TX, UK; daniel.green@liverpool.ac.uk; 3 Faculty of Medicine, University of Southampton, Southampton SO16 6YD, UK; a.j.cree@soton.ac.uk (A.C.); A.J.Lotery@soton.ac.uk (A.L.); 4 Laboratoire de Génétique Médicale INSERM U1112, Institut de Génétique Médicale d’Alsace (IGMA), Université de Strasbourg, 67000 Strasbourg, France; 5 Service de Génétique Médicale, Centre de Référence pour les Affections Rares en Génétique Ophtalmologique (CARGO), Institut de Génétique Médicale d’Alsace (IGMA), 67000 Strasbourg, France

**Keywords:** age-related macular degeneration, epigenetic clock, DNA methylation, ageing, retinal pigment epithelium, whole blood

## Abstract

DNA methylation age (DNAm age) estimation is a powerful biomarker of human ageing. To date, epigenetic clocks have not been evaluated in age-related macular degeneration (AMD). Here, we perform genome-wide DNA methylation analyses in blood of AMD patients with a documented smoking history (14 AMD, 16 Normal), identifying loci of differential methylation (DML) with a relaxed *p*-value criterion (*p* ≤ 10^−4^). We conduct DNAm age analyses using the Horvath-multi tissue, Hannum and Skin & Blood epigenetic clocks in both blood and retinal pigment epithelium (RPE). We perform Ingenuity Pathway Analysis Causal Network Analysis (IPA CNA) on the topmost significantly differentially methylated CpG probes in blood and RPE. Results show poor performance of epigenetic clocks in RPE. Epigenetic age acceleration (EAA) was not observed in AMD. However, we observe positive EAA in blood of smokers, and in smokers with AMD. DML analysis revealed hypomethylation at cg04953735 within *RPTOR* (*p* = 6.51 × 10^−5^; Δβ = −11.95%). IPA CNA in the RPE also identified *RPTOR* as the putative master regulator, predicted to be inhibited in AMD. In conclusion, this is the first study evaluating an association of epigenetic ageing in AMD. We posit a role for *RPTOR* as a common master regulator of methylation changes in the RPE in AMD.

## 1. Introduction

Ageing is a complex physiological process characterised by progressive loss of tissue functionality and an increased risk of death [1]. DNA methylation (DNAm) is the most widely studied epigenetic modification in ageing [2]. Epigenetic clocks, which estimate chronological age based on DNA methylation age (DNAm age) are promising biomarkers of human ageing, designed around well-established patterns of DNAm changes over an individual’s lifespan [2,3,4,5]. Several epigenetic clocks have been developed [6]. With few exceptions, most individual CpG probes selected for the epigenetic clocks have poor to moderate DNAm age predictive performance [7]. However, the weighted combination of many CpG probes, usually 300–400, results in highly accurate single and pan tissue DNAm age predictors [7]. Horvath’s multi-tissue [3], Hannum’s [4], and the Skin & Blood epigenetic clocks [8] were constructed using penalised multivariate elastic net regression models that select CpG probes for each respective clock by minimising the residual error of predicting age while removing redundant coefficients from the model. Importantly, they differ based on tissue types used in clock training data [3,4,8]. From a biological perspective, the epigenetic clocks are believed to reflect the function of the “epigenetic maintenance system” (EMS), a measure of cumulative work used to maintain epigenetic stability in the genome [3]. A positive or negative deviation of estimated DNAm age from chronological age is termed “epigenetic age acceleration” (EAA). Positive EAA is physiologically necessary early in life, when rapid cellular development demands that epigenetic stability is maintained [7]. Positive EAA is also reported in individuals with age-related neurodegenerative diseases including Alzheimer’s [9] and Parkinson’s disease [10], in addition to some cancers [11]. However, significant negative EAA has been identified in the cerebellum of extremely elderly humans (>100 years old) and in certain cancers [12,13,14,15], acting as a predictor of poor prognostic outcomes in the latter [15]. Together these studies indicate that both positive and negative accelerations of epigenetic age represent cellular adaptive responses to genomic/epigenomic instability, contributors to age-related disease [7].

Age-related macular degeneration (AMD) is a degenerative disease affecting the retinal pigment epithelium (RPE) and retina, representing a foremost cause of blindness with global projection estimates of 288 million cases by 2040 [16,17,18]. The greatest non-modifiable risk factor for AMD is advanced age [16], whereas smoking is the most significant modifiable risk factor [19,20,21,22]. Studies investigating a role for DNAm in AMD to date have primarily focused on site and region specific DNAm changes and their association with altered transcriptional profiles of individual genes [23,24,25,26,27]. Smoking as well as ageing exert direct effects on DNAm, with established DNAm changes occurring at specific CpG probes in blood of smokers [28,29]. Positive EAA calculated using Horvath’s multi tissue clock has also been observed in lung tissues of smokers [30]. Therefore, major AMD risk factors, such as ageing and smoking, influence epigenetic ageing and EMS responses. However, to date, analysis of epigenetic ageing using the epigenetic clocks has not been applied to primary sites of pathogenesis in AMD including RPE, or even blood samples of patients with AMD. In addition, a link between smoking status and epigenetic ageing in AMD has not been investigated.

Epigenetic age estimations and DNAm signatures are often tissue-type dependent [12,31]. In this study, we investigate a role for epigenetic ageing in AMD by performing DNAm age estimations using the Horvath multi-tissue [3], Hannum [4], and Skin & Blood [8] epigenetic clocks on age-matched RPE and whole blood from patients with AMD and healthy controls. The Horvath’s multi-tissue and Skin & Blood epigenetic clock were chosen due to favourable cross-tissue performance [3,8], and Hannum’s clock because it was constructed using solely whole blood-derived data from a cohort spanning a large age range (19–101 years) [4]. All three were chosen based on alleged robustness of chronological age prediction [3,4,8]. Firstly, we assess the reliability of different epigenetic clocks in the RPE and blood. Secondly, we analysed EAA in Normal and AMD affected RPE and whole blood. We also conducted a secondary analysis assessing an association of smoking status with EAA in AMD whole blood. Furthermore, we conducted Ingenuity Pathway Analysis Causal Network Analysis (IPA CNA) in age-matched Normal and AMD-affected RPE and whole blood. We posit a role for *RPTOR*, which encodes the Regulatory Associated Protein of mTOR Complex 1 (Raptor) as a common master regulator of methylation changes in the RPE in AMD, which also displays differential methylation in whole blood of AMD patients.

## 2. Results

### 2.1. Epigenetic Clocks Display Poor Performance in the RPE

**Figure 1:**
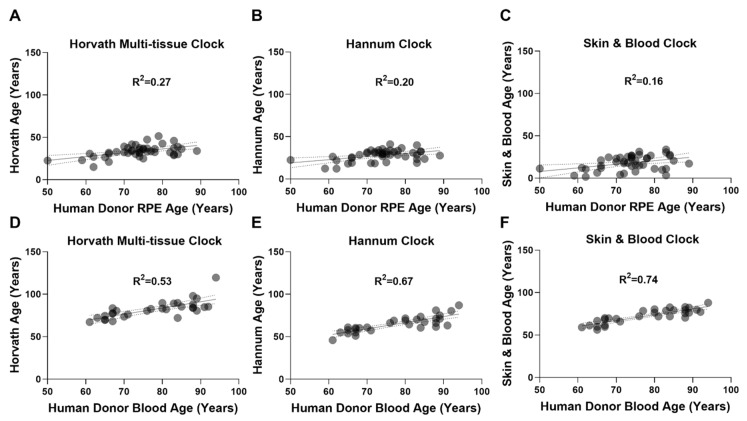
Epigenetic clocks perform poorly in RPE. Linear regressions of estimated DNAm age (years) with chronological age (years) were performed on (**A**–**C**) RPE (*n* = 44) and (**D**–**F**) whole blood-derived (*n* = 30) gDNA samples, to assess the performance of (**A**,**D**) Horvath’s multi-tissue clock, (**B**,**E**) Hannum’s clock, and (**C**,**F**) the Skin & Blood clock. (**A**–**C**) In RPE gDNA, linear regressions of DNAm age estimation with chronological age are shown for: (**A**) Horvath’s multi-tissue clock (R^2^ = 0.27); (**B**) Hannum’s clock (R^2^ = 0.20); and (**C**) Skin & Blood clock (R^2^ = 0.16). All demonstrated poor clock performance and weak correlations. In whole blood-derived gDNA, linear regressions of DNAm age estimation with chronological age for (**D**) Horvath’s multi tissue clock (R^2^ = 0.53), (**E**) Hannum’s clock (R^2^ = 0.67), and the (**F**) Skin & Blood clock (R^2^ = 0.74) demonstrated improved performance with the Skin & Blood clock showing the most accurate predictions.

We performed DNAm age estimation analysis on RPE and whole blood-derived genomic DNA (gDNA) using the Horvath multi-tissue, Hannum, and Skin & Blood epigenetic clocks. RPE DNAm data was acquired from our previously described cohort (*n* = 44) using the Illumina Infinium HumanMethylation450K BeadChip array (450K-array) [27]. Whole blood-derived gDNA methylation data was acquired using the Illumina Infinium MethylationEPIC BeadChip array (EPIC-array) from the Southampton Cohort (*n* = 30). DNAm age estimations for all clocks assessed can be found in Supplementary Materials: Additional File S1. In RPE, Horvath’s multi-tissue (Figure 1A), Hannum’s (Figure 1B), and the Skin & Blood epigenetic clocks (Figure 1C) all performed poorly. Weak positive correlations were observed between DNAm age and chronological age. In whole blood-derived gDNA, Horvath’s multi-tissue (Figure 1D), Hannum’s (Figure 1E), and the Skin & Blood clocks (Figure 1F) demonstrated better correlation between DNAm age and chronological age compared to RPE. This suggests both improved predictive capability and better calibration in whole blood. The Skin & Blood clock (Figure 1F) displayed the most accurate DNAm age estimation of all three clocks assessed.

### 2.2. Epigenetic Age Acceleration Is Not Associated with AMD

**Figure 2:**
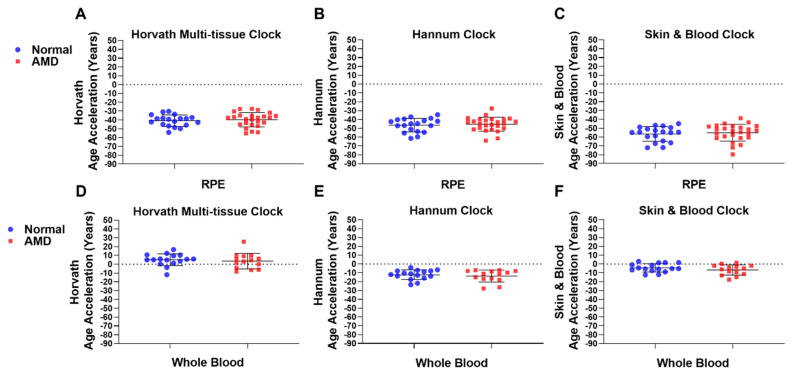
Epigenetic age acceleration is not associated with AMD. EAA analysis was conducted on AMD and Normal samples in (**A**–**C**) RPE (AMD: *n* = 25; Normal: *n* = 19) and (**D**–**F**) whole blood-derived gDNA (AMD: *n* = 14; Normal: *n* = 16) using (**A**,**D**) Horvath’s multi-tissue clock; (**B**,**E**) Hannum’s clock; and the (**C**,**F**) Skin & Blood clock. No significant difference in EAA was found for RPE using (**A**) Horvath’s multi-tissue clock (*p* = 0.5109); (**B**) Hannum’s clock (*p* = 0.7784); and the (**C**) Skin & Blood clock (*p* = 0.5733), or in whole blood-derived gDNA using (**D**) Horvath’s multi-tissue clock (*p* = 0.3769); (**E**) Hannum’s clock (*p* = 0.6374); or the (**F**) Skin & Blood clock (*p* = 0.2939). Statistical analysis was performed using a Mann-Whitney test.

We next investigated whether AMD patients displayed a positive or negative EAA when compared to Normal samples in both RPE [27] and whole blood-derived DNAm data. We define EAA as DNAm age minus chronological age. In human donor RPE, a marked and consistent negative mean EAA was observed in both Normal (*n* = 19) and AMD (*n* = 25) groups using Horvath’s multi-tissue (Normal; −40.85 years; AMD: −39.73 years) (Figure 2A); Hannum’s (Normal: −46.43 years; AMD: −45.38 years) (Figure 2B); and the Skin & Blood clock (Normal: −56.50 years; AMD: −55.10 years) (Figure 2C). When considering both Normal (*n* = 16) and AMD (*n* = 14) whole blood samples, Horvath’s multi-tissue clock displayed a small increase in EAA (Normal: +5.07 years; AMD: +3.53 years) (Figure 2D); whereas the Hannum (Normal: −12.21 years; AMD: −13.69 years) (Figure 2E) and Skin & Blood (Normal: −4.27 years; AMD: −6.78 years) clocks displayed decreases in EAA across groups (Figure 2F). No significant difference was observed in EAA between Normal and AMD groups across all clocks tested in the RPE (Figure 2A–C) and whole blood-derived gDNA (Figure 2D–F), suggesting no association of EAA with AMD across these tissues.

### 2.3. Smokers Display Increased Epigenetic Age Acceleration Compared to Non-Smokers

**Figure 3:**
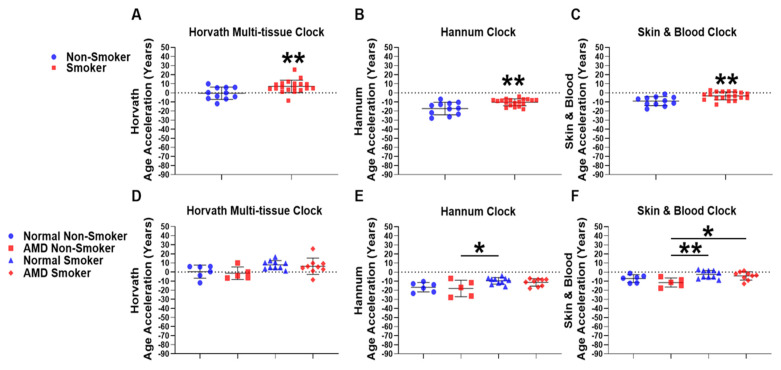
Epigenetic age acceleration is associated with smoking but not AMD in whole blood. EAA analysis was conducted using DNAm microarray data to compare differences from Smoker (*n* = 19) and Non-Smoker (*n* = 11) whole blood gDNA using the (**A**) Horvath multi-tissue, (**B**) Hannum and, (**C**) Skin & Blood epigenetic clocks. EAA analysis was further stratified to compare differences amongst the following four groups: Normal Non-Smoker (*n* = 6); AMD Non-Smoker (*n* = 5); Normal Smoker (*n* = 10); AMD Smoker (*n* = 9) using (**D**) Horvath’s multi-tissue, (**E**) Hannum’s and (**F**) the Skin & Blood epigenetic clocks. Smokers displayed a significant increase in EAA using (**A**) Horvath’s multi-tissue clock (*p* = 0.0071), (**B**) Hannum’s clock (*p* = 0.0014), and (**C**) the Skin & Blood clock (*p* = 0.0025) when compared to Non-Smoker groups. Following stratification into four separate groups, no significant differences were observed following one-way ANOVA when analysing (**D**) Horvath’s multi-tissue epigenetic clock, but were identified using the (**E**) Hannum and (**F**) Skin & Blood epigenetic clocks (Supplementary Materials: Additional File S2, Table S5). Following application of the TukeyHSD post-hoc test, no significant differences were identified when comparing Normal Non-Smoker and AMD Non-Smoker, in addition to Normal Smoker and AMD Smoker groups using the (**E**) Hannum and (**F**) Skin & Blood epigenetic clocks. Significant differences were identified when comparing EAA between AMD Non-Smokers and Normal Smokers using the (**E**) Hannum and (**F**) Skin & Blood epigenetic clocks. When comparing AMD Non-Smoker and AMD Smoker groups, no significant difference was found when comparing the (**E**) Hannum epigenetic clock, however a significant increase in EAA was found using the (**F**) Skin & Blood epigenetic clock (for individual *p*-values, see Supplementary Materials: Additional File S2, Table S6).Statistical analysis was performed using (**A**–**C**) an unpaired *T*-test of means and an (**D**–**F**) Ordinary one-way ANOVA followed by the (**E**,**F**) TukeyHSD post-hoc test. (* *p ≤* 0.05). (** *p ≤* 0.01).

Smoking is the most significant modifiable risk factor for AMD [19]. Information on smoking status was available for whole blood-derived gDNA samples from the Southampton Cohort. Therefore, we investigated the association of smoking with EAA in whole blood-derived gDNA using univariate analysis comparing Smokers and Non-Smokers. We found significant increases in EAA in Non-Smoker compared to Smoker groups using the Horvath multi-tissue epigenetic clock (Non-Smoker: −0.43 years; Smoker: +7.12 years) (Figure 3A). Using the Hannum (Non-Smoker: −17.26 years; Smoker: −10.38 years) (Figure 3B) and Skin & Blood (Non-Smokers: −9.11 years; Smoker: −3.32 years) (Figure 3C) epigenetic clocks, we found larger mean negative EAAs across groups but still observed significant increases in EAA when comparing Non-Smoker and Smoker groups.

### 2.4. AMD Smokers Display Positive Epigenetic Age Acceleration Using the Skin & Blood Epigenetic Clock

We next investigated whether presence of AMD was associated with EAA in Smokers and Non-Smokers, and whether smoking status was associated with EAA in AMD or Normal samples using whole blood-derived gDNA. We stratified our analysis to compare the following four groups: Normal Non-Smoker (*n* = 6); AMD Non-Smoker (*n* = 5); Normal Smoker (*n* = 10); and AMD Smoker (*n* = 9), using the Horvath multi-tissue (Figure 3D), Hannum (Figure 3E), and Skin & Blood (Figure 3F) epigenetic clocks, respectively. Individual *p*-values for each respective comparison across all clocks tested are listed in Supplementary Materials: Additional File S2, Tables S5 and S6.

Firstly, we performed a one-way ANOVA for the Horvath multi-tissue (Figure 3D), Hannum (Figure 3E) and Skin &Blood (Figure 3F) epigenetic clocks. A Benjamini–Hochberg FDR correction was applied to F-test values for each one-way ANOVA (Supplementary Materials: Additional File S2, Table S5). The Horvath multi-tissue clock did not display significant FDR-adjusted *p*-values (Figure 3D). However, the Hannum (Figure 3E) and Skin & Blood (Figure 3F) epigenetic clocks did display significant FDR-adjusted *p*-values and were selected for the TukeyHSD post-hoc test to assess differences across groups. Details of the one-way ANOVA with the applied TukeyHSD post-hoc test are found in Additional File S2, Table S6. Significant differences were identified in EAA between AMD Non-Smokers and Normal Smokers using the Hannum (* *p* = 0.0334, mean year difference: −8.42 years) and Skin & Blood clocks (** *p* = 0.0064, mean year difference: −8.94 years). However, we did not observe any differences in EAA between Normal Non-Smoker compared to AMD Non-Smoker; and Normal Smoker compared to AMD Smoker groups using the Hannum (Figure 3E) and Skin & Blood (Figure 3F) epigenetic clocks, suggesting that disease state is not associated with positive or negative EAA in Smokers. We next sought to assess whether smoking was associated with EAA in Normal and AMD patients. Normal Smoker patients did not display any increase in EAA compared to Normal Non-smokers. However, in AMD patients, smoking was associated with a significant positive EAA when compared to Non-Smokers in AMD using the Skin & Blood epigenetic clock (* *p* = 0.0335, mean year difference: −7.347 years). (Figure 3E,F).

### 2.5. Differentially Methylated CpG Probes Identified in Whole Blood gDNA from AMD Patients

We next investigated DNAm differences at individual CpG probes in AMD (*n* = 14) and Normal (*n* = 16) whole blood-derived gDNA using DNAm data generated by the EPIC-array. We conducted differentially methylated locus (DML) and differential region (DMR) analysis between AMD and Normal samples. All AMD samples used in our analysis from the Southampton Cohort were AREDS grade 2 (78%) or grade 3 (22%). No significant CpG probes or regions were identified in our cohort following FDR multiple testing correction in our DML or DMR analysis [32]. However, applying an unadjusted *p*-value cut-off criterion of *p* ≤ 10^−4^ [27], we identified 21 differentially methylated CpG probes between AMD and Normal groups (Table 1). We identified five promoter based CpG probes, 14 gene body based CpG probes, and two intergenic probes. Across the top 21 variable probes (*p* ≤ 10^−4^), we observed low mean β-value differences between AMD and Normal samples (Δβ) (Δβ ± ≤5%) with two CpG probes displaying large effect sizes (Δβ ± ≥10%). The largest effect size was identified in *RPTOR* (cg04953735, Intron 3, Δβ = −11.95%). For a scatter plot representing methylation β-values, please see Supplementary Materials: Additional File S2, Figure S1. cg04953735 within *RPTOR* co-localised with H3K4me1 and H3K27ac enrichment using layered data in seven cell lines from ENCODE, UCSC genome browser, suggestive of methylation differences occurring within an active enhancer region. In addition, cg04953735 lies within a predicted 2032bp enhancer region (GeneHancer: GH17J080678) within intron 3 of *RPTOR*, 134008bp upstream of a predicted interaction region within the TSS200 of *RPTOR.*

### 2.6. Ingenuity Pathway Causal Network Analysis Identified RPTOR as a Master Regulator of Methylation Changes in RPE

**Figure 4:**
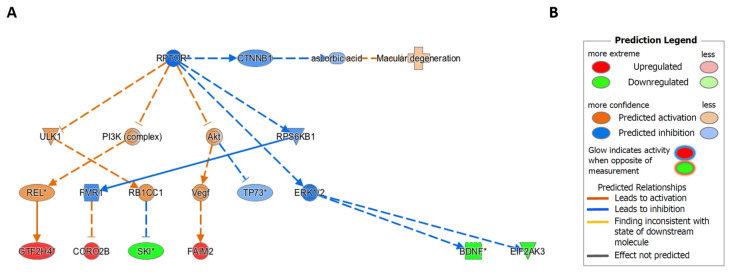
Ingenuity Pathway Analysis Causal Network Analysis identifies *RPTOR* as a master regulator for DNA methylation changes in AMD RPE. (**A**) Ingenuity Pathway Analysis Causal Network Analysis was performed on the top most differentially methylated probes identified by the 450K-array in AMD human RPE gDNA samples. *RPTOR* inhibition was identified as a master upstream regulator of *SKI*, *GTF2H4*, *CORO2B*, *FAIM2*, *BDNF*, and *EIF2AK3. RPTOR* inhibition was also found to inhibit *CTNNB1*, which inhibits ascorbic acid, predicted to activate the term “macular degeneration” in IPA. (**B**) Prediction legend for the Ingenuity Pathway Analysis Causal Network Analysis.

We performed moderated *t*-tests using limma and conducted IPA CNA on the top 301 most significantly differentially methylated CpG probes in blood, based on a *p*-value threshold of *p* ≤ 10^−3^ between AMD and Normal groups. Following filtering of CpG probes in intergenic areas and assigning multiple CpG probes mapping to a single gene as one single gene, the IPA CNA in blood revealed 212 genes for analysis. Supplementary Materials: Additional File S3, demonstrates the full results of IPA CNA for the top most differentially methylated CpG probes between AMD and Normal samples in blood. We also assessed the top most variable probes from our published genome wide DNA methylation dataset obtained from RPE [27]. We performed IPA CNA by scoring against the disease terms “macular degeneration” (Figure 4). No master regulators were identified among the top 301 most significantly differentially methylated probes (*p* ≤ 10^−3^) using whole blood-derived gDNA from the Southampton Cohort that scored against the term “macular degeneration” (Additional File S3). However, in the RPE, IPA CNA identified inhibition of *RPTOR* (Figure 4) as master regulator for methylation changes amongst the top most significantly differentially methylated CpG probes that scored against the term “macular degeneration” in AMD RPE [27]. Full results of IPA CNA for the top most significantly differentially methylated CpG probes between AMD and Normal samples in RPE, are shown in Supplementary Materials: Additional File S3. In addition, the predicted inhibition of *RPTOR* in AMD RPE leads to the predicted inhibition of *CTNNB1* encoding beta-catenin, predicted to activate the disease term “macular degeneration”.

## 3. Discussion

This is the first study to our knowledge formally evaluating whether EAA is associated with AMD and important risk factor covariates including smoking status. We sought to address whether EAA is observed in the RPE [27], as it is a primary site of AMD pathogenesis, and in whole blood, as the epigenetic clocks have been widely applied and validated in blood-derived gDNA.

In the RPE, we observed a marked negative EAA across all groups with no significant differences in EAA between AMD and Normal samples using all three clocks. This result cannot be characterised as true negative age acceleration because of poor performance of the epigenetic clocks in RPE [33]. The consistent poor correlation of predicted DNAm age with chronological age observed in the RPE markedly improved when analysing whole blood-derived gDNA data, explained by the datasets used to train each respective epigenetic clock [3,4,8]. Although we might expect any non-zero EAA in the RPE to also be associated with weak correlation, our findings are key in illustrating the potential biological and technical deviations present when assaying RPE samples as opposed to whole blood using the epigenetic clocks. Non-zero deviations do indeed have an impact on the accuracy and correlation of the estimates; however, we posit that the epigenetic age is always underestimated in RPE rather than errors being positively and negatively distributed around the mean. This suggests clock calibration issues in RPE or a currently unknown biological phenomenon, although the better predictive capacity of the clocks in blood supports the former assumption. As such, we believe the poor accuracy of epigenetic clocks in RPE is a key finding, especially in the context of age-dependent hypermethylation of *ELOVL2* in ageing ocular tissues increasingly referenced as a reliable biomarker [4,8,34]. The data provided welcomes the generation of multivariate models to further delineate the complexity of ageing in RPE or address the current calibration issues with existing clocks.

Reasonable performance of each respective epigenetic clock in whole blood, however, strengthens the observation of no association of EAA with AMD in blood, though this remains open to further investigation in the RPE, which can be addressed using a bespoke RPE epigenetic clock with greater predictive accuracy. The highly accurate skeletal muscle [35] and breast tissue [36] epigenetic clocks have set precedents to this approach. These clocks share little overlap in CpG probe selection with Horvath’s multi-tissue clock suggesting that bespoke clocks capture separate tissue specific epigenetic ageing processes [36]. Therefore, construction of a tissue-specific RPE clock is necessary for future studies to capture the specific epigenetic ageing processes in the RPE.

A strength of this study was having information regarding the smoking status of AMD patients from which blood samples were taken, allowing stratified analyses to investigate whether smoking was associated with EAA in AMD. Smoking, the greatest modifiable risk factor for AMD, substantially increases AMD relative risk (RR) with RR of disease varying from 2.7 to 6.6 in current smokers in unrelated mixed-gender cohorts [19,20,21,22]. In accordance with previous studies examining EAA using Horvath’s multi-tissue clock on DNAm data from lung tissue of smokers [30], we observed a significant positive EAA in all three epigenetic clocks tested. The three clocks are predictors of chronological ageing and display a degree of CpG probe overlap [6], however each clock captures facets of a diverse, often tissue-type specific ageing process [6]. Taken together, our results suggest that smoking exerts a significant and broad effect on DNAm age in blood and further regulates biological ageing across several tissue types. To confirm whether this effect is present in the RPE, future studies will require smoking status documentation.

Using the Skin & Blood clock, which demonstrated the greatest predictive accuracy for chronological age in our whole blood samples, we found significant positive EAA in AMD Smokers compared to Non-Smokers, a finding not replicated in the Horvath multi-tissue [3] and Hannum epigenetic clocks [4]. One explanation for this may lie in confounding factors affecting clock performance based on the age range of patient samples used in our study, which included RPE/blood from patients > 50 years old. Previous studies have demonstrated failure of both the Horvath multi-tissue and Hannum epigenetic clocks to accurately predict chronological age in older cohorts [12,33,37]. It is proposed that this occurs due to methylation saturation (i.e., select CpG probes reaching either 0 or 100% methylation later in life), in addition to confounding from other age related processes [33]. In the case of Horvath’s multi-tissue epigenetic clock, poor predictive capability in elderly samples can be partially attributed to lower representation of tissues from elderly individuals in test data [33]. Inaccurate prediction of DNAm age in elderly individuals has also been shown to be a facet of Hannum’s clock [33], where test data individuals were aged between 19–101 years [4]. Therefore, the reasons for less precise age prediction in older individuals using the Hannum/Horvath clock are not entirely clear. However, previous poor performance in older datasets for the Horvath multi-tissue and Hannum clocks may explain why the Skin & Blood clock performed best out of all three clocks tested in blood-derived gDNA. While the Skin and Blood clock has also shown a degree of error in elderly samples due to age confounding [37], it consistently outperforms predictive capacity in blood samples when compared to Horvath’s multi tissue clock and Hannum’s clock, a finding confirmed in this study [7,37]. Therefore, our observation that smokers with AMD exhibit accelerated epigenetic ageing compared to Non-Smokers with AMD is strengthened by identifying this effect in the clock with the greatest predictive accuracy for the demographic age and tissue type represented in our dataset.

To support our analyses of epigenetic ageing in AMD, we performed DML analysis in AMD whole blood-derived gDNA. In line with previous microarray-based studies in blood of patients with AMD [26], we were unable to identify significantly differentially methylated CpG probes following FDR-adjusted multiple testing correction. However, using a *p*-value cut-off criterion of *p* ≤ 10^−4^ [27], we identified 21 differentially methylated CpG probes, with no overlap between our top 21 most variable probes and those observed in other genome-wide studies [26,27]. Small effect size was observed amongst our top most variable probes (Δβ ≤ 5%), consistent with the effect sizes reported in the literature [23,26]. We propose that this effect is similar to the ageing process outlined by Horvath’s multi-tissue clock, whereby DNAm levels at individual CpG probes correlate poorly with age, but the composite effect of a larger number of probes represents consistent and reproducible epigenetic changes occurring during the ageing process [3]. Collectively, our finding suggests that a composite effect of small methylation changes at a number of CpG probes drives early and intermediate AMD, representing a more accurate picture of DNAm changes occurring during AMD development [3,38].

We next investigated a causal relationship for methylation changes using IPA CNA at the top 301 most significantly differentially methylated CpG probes in blood and top significantly differentially methylated CpG probes in the RPE, identified in our previous study [27]. IPA CNA may detect novel master upstream regulators acting through intermediate downstream regulators affecting gene expression [39], in this case using directional log-fold methylation changes as a proxy for expression changes. IPA CNA creates networks based on known interactions within the IPA knowledge base [39]. While the causal relationship of methylation changes between genes is less defined than that of gene expression differences, IPA CNA can be used to identify master regulators [39]. IPA CNA maps differentially methylated probes to genes, and then maps these genes to disease states, such as AMD. However, previous studies identified bias in gene-set analysis applied to high throughput DNAm data [40]. The reason for this is the key assumption in GSA that genes have, *a priori*, the same probability of appearing in the list regardless of experimental condition [40]. In the case of DNAm data, this can lead to bias due to differences in the prevalence of CpG probes associated with different genes [40]. IPA accounts for this bias by recognising a gene that appears multiple times in a list only once. In this context, we were unable to identify master regulators of genes of differentially methylated CpG probes in our top 301 significantly differentially methylated CpG probes in whole blood, however we identified inhibition of *RPTOR* as the putative upstream master regulator in AMD RPE [27].

Importantly, in blood, we also identified the largest methylation change of any probe within *RPTOR*, with a substantial decrease in methylation (Δβ = −11.5%; *p* = 6.51 × 10^−5^). Raptor is an adaptor protein involved in regulating the activity of Mammalian Target of Rapamycin Complex-1 (mTORC1), by facilitating recruitment of substrates to the mTOR kinase [41] which regulates ageing, cellular growth, stress responses and inhibits autophagy [42]. mTOR plays a central role in ageing and is implicated in proteostasis, mitochondrial function and cellular senescence [43]. Raptor inhibition has been shown in human donor AMD RPE concurrent with increased mTOR activation [42] a finding corroborated by previous evidence showing that primary RPE cell cultures from elderly individuals display increased mTORC1 activity [44]. Of further relevance, overactive mTORC1 has been shown to indirectly downregulate the expression of protein kinase ER-like Kinase (PERK), a key component of the unfolded protein response, in mouse embryonic fibroblasts [45]. *EIF2AK3*, the gene encoding PERK, displays significant downregulation in AMD RPE [27]. Together, these findings support key roles for upregulated mTORC1 activity and inhibition of *RPTOR* in AMD, although further experimental evidence is required to confirm this association.

We acknowledge that whilst there is added utility in the assessment of epigenetic ageing in primary affected disease tissues such as RPE in AMD, epigenetic ageing processes in peripheral tissues, such as blood, may still provide useful insights to generate a systemic ageing profile associated with AMD. In this context, evidence suggests that although AMD is primarily an ocular disease, systemic factors contribute to disease development [26,46]. The growing body of evidence identifying DNAm changes both in RPE [27] and in blood [26] of AMD patients is important as altered epigenetic maintenance processes implicated in ageing and ageing-related diseases, may potentially be reversed. Lu et al. recently demonstrated that epigenetic ageing is not a unidirectional process, as induced ectopic expression of the *OCT4, SOX2* and *KLF4* genes in mouse retinal ganglion cells reversed vision loss in mouse models of glaucoma and ageing, associated with restoration of a youthful DNAm age [47].

A limitation of this study lies in the sample size used to investigate differences in methylation between AMD and Normal whole blood-derived gDNA with insufficient power to detect FDR-adjusted differentially methylated CpG probes. This may also underlie the absence of EAA associated in AMD. However, a previous EWAS investigating DNAm differences in whole blood failed to identify significant differential methylation following FDR-adjustment using a significantly increased cohort size (AMD, *n* = 198; Control, *n* = 100) [26]. Therefore, future studies investigating both differential methylation and EAA will require greater samples sizes [26,27].

In conclusion, our findings of positive EAA using all three epigenetic clocks in Smokers, and in Smokers with AMD using the most appropriate Skin & Blood epigenetic clock represents an important avenue for further development [47]. Furthermore, our identification of *RAPTOR* as master regulator of DNAm changes in AMD RPE supports its role in both ageing and AMD and provides a key target for future functional studies.

## 4. Materials and Methods

### 4.1. Sample Collection, Grading and DNA Extraction

Peripheral whole blood samples and gDNA were extracted from individuals within the Southampton Case-Control Cohort (referred to as the “Southampton Cohort”) in a manner described previously [48]. Demographic information was provided including age (years), gender (male or female), and smoking status (Smoker or Non-Smoker) (Supplementary Materials: Additional File S2, Table S1). Age-matching statistics for the purpose of this study comparing AMD and Normal groups, in addition to Non-Smoker and Smoker groups, can be found in Supplementary Materials: Additional File S2, Tables S3 and S4. Samples were obtained from individuals phenotyped according to the Age-Related Eye Disease Study (AREDS) classification (Additional File S2: Table S2) [49]. Samples from patients exhibiting advanced AMD were excluded from the study. A total of 30 patient samples including 16 Normal, 3 AREDS grade 2 (early AMD) and 11 AREDS grade 3 (intermediate AMD) (AMD total, *n* = 14) were selected (Supplementary Materials: Additional File S2, Table S1). Analysis of peripheral whole blood gDNA degradation levels were assessed prior to bisulfite conversion for methylation analyses by performing gel electrophoresis in a previously described manner [27].

### 4.2. Illumina Infinum MethylationEPIC BeadChip Array

DNAm levels were measured using the EPIC-array (Illumina Inc., San Diego, CA, USA), interrogating 865 918 CpG sites covering ≥99% of RefSeq genes. Samples run on the EPIC-array were randomized and balanced for disease status and smoking status to minimise chip and row specific effects. The EPIC-array was conducted at the Edinburgh Clinical Research Facility (Edinburgh, UK) incorporating technical controls into the experimental design. In total, 500 ng (50 ng/μL) peripheral whole blood-derived gDNA was bisulfite converted using the EZ-96 DNA methylation kit (Zymo Research, Irvine, CA, USA) and hybridised to the EPIC-array according to the manufacturer’s instructions. Quality control analysis was performed using GenomeStudio (v2011.1). Raw IDAT files were then read into *R* (version 3.31) using the *read.metharray.exp* function within the *minfi* package [50] The dataset and analysis of the Southampton Cohort has been deposited in ArrayExpress with submission number: E-MTAB-11279.

### 4.3. Pre-Processing and Normalisation

EPIC-array data was analysed using functionality within the *minfi* package [50]. Each sample was subjected to various quality control measures. Cell type proportions were corrected using the estimateCellCounts2 function in R. Briefly, “CD8T”, “CD4T”, “NK”, “Bcell”, “Mono”, and “Neu” were deconvoluted from our mixed whole blood samples [51]. Furthermore, samples were checked for global hybridisation quality based on an average probe detection *p*-value threshold of *p* ≥ 0.05. Samples not meeting these criteria were removed from the analysis. Individual CpG probes exceeding a detection *p*-value of *p* ≥ 0.01, indicating a failed position, were removed from the analysis. Probes located on chromosomes X & Y and probes within two base pairs of a single nucleotide polymorphism (SNP) with a minor allele frequency ≥0.05 were also removed, as well as probes previously found to cross hybridise to multiple genomic locations [52,53]. Samples were normalised using the Subset-quantile Within Array Normalisation (SWAN) algorithm to correct for biases between type I and type II probe distributions [54]. Following filtering, 784,486 CpG probes were available for downstream analysis.

### 4.4. Epigenetic Clock Analyses

DNAm age was calculated on DNAm data obtained from whole blood-derived gDNA of Normal and AMD patients from the Southampton Cohort, analysed using the EPIC-array, and DNAm data from ocular tissue (human RPE) of Normal and AMD patients from our previously described cohort of individuals of European descent, analysed using 450K-array data (accessed from ArrayExpress: E-MTAB-7183) [date accessed: 24 May 2020]. [27]. DNAm age estimations were performed using Horvath’s multi-tissue [3], Hannum’s [4], and the Skin & Blood epigenetic [8] clocks using a publicly available online calculator (available at: http://dnamage.genetics.ucla.edu/, accessed on 9 September 2021). For outputs of all epigenetic clock data in whole blood and RPE-derived gDNA, please see Supplementary Materials: Additional File S1. We assessed the performance of each respective clock on DNAm data derived from RPE gDNA (*n* = 44) using 450K-array data from our previously described cohort [27]. We also assessed the performance of these three clocks using whole blood-derived (*n* = 30) gDNA methylation data acquired by EPIC-array from the Southampton Cohort. Performance was assessed using linear regression of estimated DNAm age with chronological age of patient-derived samples from each respective tissue type. EAA was calculated as the difference between estimated DNAm age and chronological age (DNAm age-chronological age). Assessment for normal distribution of data was performed using a Shapiro–Wilks Test for Normality prior to conducting comparisons using statistical tests. Normality tests were passed following α > 0.05. Non-parametric Mann–Whitney tests comparing EAA of whole blood-derived gDNA samples and RPE-derived gDNA samples were performed across the groups: “AMD vs Normal” in whole blood and RPE. Univariate analyses employing an unpaired *t*-test comparing EAA was performed for: “Smoker vs Non-Smoker” groups. We stratified our cohort into the following groups: Normal Non-Smoker (*n* = 6); AMD Non-Smoker (*n* = 5); Normal Smoker (*n* = 10); and AMD Smoker (*n* = 9). We computed ANOVAs for each respective epigenetic clock. We applied the FDR correction to the *F* Test values of each ANOVA and, if significant following FDR adjustment, applied the TukeyHSD post-hoc test to determine significant differences between groups (Supplementary Materials: Additional File S2, Tables S5 and S6). All statistical analysis was performed using *R* (version 3.31) and GraphPad Prism (ver 8.0.2, GraphPhad Software Inc., San Diego, CA, USA).

### 4.5. Differentially Methylated CpG Probe and Region Analysis

To identify differentially methylated CpG probes, univariate statistical analysis for each CpG probe was performed using the linear models for microarray data (*Limma*) package [55]. To implement the linear models, we built a contrast matrix with coefficients for “AMD” and “Normal”. Significance was determined based on a Benjamini–Hochberg (BH)-adjusted False Discovery Rate (FDR) ≤ 0.05. In a scenario where no differentially methylated CpG probes were found using a BH-adjusted FDR ≤ 0.05, an unadjusted *p*-value criterion of *p* ≤ 10^−4^ was applied [27]. We assessed for differentially methylated regions using *DMRcate* [56]. Regions were defined as blocks of 1000 nucleotides fitting a gaussian kernel smoothed function. We considered a region to be differentially methylated if its BH-corrected FDR was ≤0.1 and had an absolute mean beta fold change >0.1 (10%)).

### 4.6. Histone Modification Enrichment and GeneHancer Analysis

For the top most variable probes in whole blood DNAm data meeting the unadjusted *p*-value criterion of *p* ≤ 10^−4^, analysis of histone modification enrichment was performed using the overlaid H3K4Me1, H3K4Me3, and H3K27Ac track functions within the Integrated Regulation track from the UCSC genome browser (available from: https://genome.ucsc.edu/cgi-bin/hgTrackUi?g=wgEncodeReg, accessed on 5 August 2021). The layered H3K4Me1, H3K4Me3, and H3K27Ac tracks consist of Chromatin Immunoprecipitation Sequencing data from seven cell lines: GM12878, H1-hESC, HSMM, HUVEC, K562, NHEK, and NHLF. GeneHancer analysis was performed using the Enhancer and Promoter functions from GeneHancer track within the UCSC genome browser (available from: https://genome.ucsc.edu/cgi-bin/hgTrackUi?db=hg19&g=geneHancer, accessed on 5 August 2021) to identify putative enhancer and promoter elements signatures. Additionally, interactions between predicted enhancers and regions within target genes were analysed using the Interaction function within the Enhancer and Promoter from GeneHancer track.

### 4.7. Ingenuity Pathway Analysis

The Causal Network Analysis (CNA) function within Ingenuity Pathway Analysis (IPA) was used to construct networks to investigate novel upstream regulators associated with AMD. Both direct and indirect relationships were considered in the general settings. All node types and data sources were considered, and confidence gained using experimentally observed and high (predicted) interactions. Interactions were considered from available mammals (human, mouse, and rat). All tissue lines and mutations were considered. CNA was performed against the disease term “macular degeneration” for the variable probes from both respective datasets (RPE and blood) to identify master regulators based on activation z-scores (z ≥ 2.0). This was performed on the top most differentially methylated probes that met the *p*-value criterion (*p* ≤ 10^−6^) in the RPE of patients with AMD, as published in [27] (Additional File S3); in addition to the top-301 variable probes (*p* ≤ 10^−3^) identified in blood-derived gDNA in patients with AMD (Additional File S3).

## Figures and Tables

**Table 1:** Differentially methylated CpG probes identified in whole blood of patients with AMD using a relaxed *p*-value criterion (*p* ≤ 10^−4^).

Probe I.D.	Gene I.D.	logFC	Unadjusted *p*-Value	Δβ (* ±≥10%)	Chromosome:Position (Hg19)	Relation to CpG Island	Relation to Gene
cg24522809	*UPP2*	−0.472041082	1.01 × 10^−6^	−3.35	chr2:158851594	Open Sea	TSS200
cg15985873	*NOX5*	0.41292444	1.40 × 10^−5^	2.82	chr15:69264323	Open Sea	Body (Intron)
cg21175976	*BLK*	−0.922913398	1.53 × 10^−5^	−9.49	chr8:11421337	Island	Body (Intron)
cg07212053	*UBE4A*	0.480522948	1.87 × 10^−5^	1.1	chr11:118230307	Island	Body (1st Exon)
cg14426911	*SEMA5A*	−0.637685904	2.07 × 10^−5^	−4.36	chr5:9363104	Open Sea	Body (Intron)
cg05306123	*INTS7*	0.52368943	2.60 × 10^−5^	4.33	chr1:212159068	Open Sea	Body (Intron)
cg06569202	*DBP*	0.511049469	4.21 × 10^−5^	1.42	chr19:49140842	Island	TSS200
cg12917056	*PDC*	0.545557387	4.38 × 10^−5^	4.38	chr1:186416576	OpenSea	Body (Intron)
cg12855166	*MYO1D*	−0.955212746	4.56 × 10^−5^	−0.88	chr17:30846586	Island	Body (Intron)
cg23282837	*CSMD3*	−0.554156229	5.06 × 10^−5^	−2.92	chr8:114449418	Open Sea	TSS200
cg17303711	*ZSCAN22*	0.472499092	5.27 × 10^−5^	1.68	chr19:58838235	Island	TSS200
cg17303822	*-*	1.107838488	5.40 × 10^−5^	8.89	chr4:120992550	Open Sea	Intergenic
cg22945982	*VWA8*	0.827121348	5.85 × 10^−5^	4.52	chr13:42443309	Open Sea	Body (Intron)
cg04953735	*RPTOR*	−0.731183406	6.51 × 10^−5^	−11.95 *	chr17:78652628	Open Sea	Body (Intron)
cg08636246	*RSBN1*	0.400242281	7.37 × 10^−5^	1.14	chr1:114354993	Island	Body (1st Exon)
cg17247365	*WWOX*	−0.787476578	7.97 × 10^−5^	−9.56	chr16:78275151	Open Sea	Body (Intron)
cg03380182	*-*	0.777809274	8.36 × 10^−5^	10.68 *	chr1:244065456	Open Sea	Intergenic
cg13955747	*TMEM18*	0.301564284	8.38 × 10^−5^	1.54	chr2:677585	Island	TSS200
cg22541572	*LRCH1*	0.662406112	8.71 × 10^−5^	4.64	chr13:47237047	Open Sea	Body (Intron)
cg07642595	*LAMP1*	−0.545207974	9.06 × 10^−5^	−6.63	chr13:113952548	South Shore	Body (Intron)
cg09228785	*CD82*	0.574387376	9.91 × 10^−5^	8.33	chr11:44630602	Open Sea	Body (Intron)

Differential methylation analysis of EPIC-array data from AMD (*n* = 14) and Normal (*n* = 16) whole blood-derived gDNA samples. Differentially methylated probes were identified using a relaxed *p*-value criterion (*p* ≤ 0.0001). * denotes CpG probes displaying a methylation difference of ≥10% [Δβ ≥ 10%]. Δβ (mean β value AMD—mean β value control), TSS (transcription start site), Body (gene body), and Intergenic (Intergenic region not mapping to known gene).

## Data Availability

All datasets utilised here are open-access datasets accessible on ArrayExpress and listed in Section 4.

## Supplementary Materials

The following are available online at https://www.mdpi.com/article/10.3390/ijms222413457/s1.

## Abbreviations

450K-array	Illumina Infinium HumanMethylation450 BeadChip Array
AREDS	Age-related Eye Disease Study
AMD	Age-related Macular Degeneration
DNAm	DNA methylation
DNAm Age	DNA methylation age
EAA	Epigenetic Age Acceleration
EMS	Epigenetic Maintenance System
EPIC-array	Illumina Infinium MethylationEPIC BeadChip Array
FDR	False Discovery Rate
gDNA	Genomic DNA
IPA CNA	Ingenuity Pathway Analysis; Causal Network Analysis
mTORC1	Mammalian Target of Rapamycin Complex 1
PERK	Protein Kinase ER-like Kinase
RAPTOR	Regulatory Associated Protein of mTOR Complex 1
RPE	Retinal Pigment Epithelium
SWAN	Subset-quantile Within Array Normalisation
