# Supplementary material for: Epigenetic Age Acceleration Is Not Associated with Age-Related Macular Degeneration
Source: Int J Mol Sci. 2021 Dec 15;22(24):13457. doi: 10.3390/ijms222413457 (PMC8705580; doi:10.3390/ijms222413457)
Supplement: Supplementary file 1 [file ijms-22-13457-s001.zip › Additional_File_S2.pdf]

Sample I.D.	Age when sample taken	Gender	Ever Smoked (Yes/NO)	AREDS
SC090	65	Male	Yes	Normal
SC241	65	Male	Yes	Normal
SC272	65	Male	Yes	Normal
SC054	67	Female	Yes	Normal
SC205	67	Female	Yes	Normal
SC291	67	Male	Yes	Normal
SC366	67	Female	Yes	Normal
SC137	68	Female	Yes	Normal
SC230	70	Male	Yes	Normal
SC136	71	Female	Yes	Normal
SC282	83	Male	No	Normal
SC122	84	Female	No	Normal
SC321	84	Female	No	Normal
SC105	85	Female	No	Normal
SC065	88	Male	No	Normal
SC339	89	Female	No	Normal
SA012	91	Female	No	3
SA046	88	Female	No	3
SA246	88	Female	No	3
SA292	92	Female	No	3
SA348	88	Female	No	3
SA118	76	Male	Yes	2
SA244	63	Male	Yes	2
SA378	77	Male	Yes	2
SA022	81	Male	Yes	3
SA086	61	Female	Yes	3
SA088	89	Female	Yes	3
SA151	80	Female	Yes	3
SA167	94	Male	Yes	3
SA273	80	Female	Yes	3

Table S1. Demographic Characteristics of blood gDNA samples used for whole genome DNA methylation analysis with EPIC array.

AMD Level	AREDS
1	Drusen maximum size < 63 μm and total area < 125 μm, and no pigment abnormalities
2	(a) Drusen maximum size ≥ 63 μm but < 125 μm, or<br>(b) Drusen total area ≥ 125 μm, or<br>(c) RPE abnormalities consistent with AMD
3	(a) Drusen maximum size ≥ circle 125 μm, or<br>(b) Drusen maximum size ≥ circle 63 μm and total area > 180 μm and type is soft indistinct, or<br>(c) Drusen maximum size ≥ 63 μm and total area > 660 μm and type is soft distinct, or<br>(d) Geographic atrophy (atrophy > 180 μm) within grid but none at centre of macula
4	(a) Geographic atrophy in central subfield (advanced)<br>(b) Evidence of Neovascular AMD

Table S2. AREDS Grading System.

AMD level 1: no AMD; AMD level 2: Early AMD; AMD level 3: Intermediate AMD; AMD level 4: advanced atrophic AMD (Geographic atrophy) or choroidal neovascularization. Table adapted from reference [47].

		Normal	AMD	*p*-value*
**N=44**	Average Age (Yrs±SD)	74.06±9.39 (n=16)	82±10.21 (n=14)	0.0614
	Median Age (Min-Max)	69 (65-89)	84.5 (61-94)	-
	Male (%)	7 (43%)	5 (35%)	-
	Female (%)	9 (57%)	9 (65%)	-

Table S3. Demographic characteristics of RPE gDNA samples (AMD cases and normal human donor RPE cells) analysed by genome wide DNA methylation 450k array (Porter et al. 2019). Mean and standard deviation (SD), median, minimum (min) and maximum (max) values were used to describe the distributions of age. *From a non-parametric Mann-Whitney U test of means.

		Non-Smoker	Smoker	*p*-value*
**EWAS Discovery Cohort N=44**	Average Age (Yrs±SD)	87.27±2.93 (n=11)	72.26±9.10 (n=19)	<0.0001
	Median Age (Min-Max)	88 (83-92)	68 (61-94)	-
	Male (%)	2 (18%)	10 (52%)	-
	Female (%)	9 (82%)	9 (48%)	-

Table S4. Comparison of demographic characteristics of Smokers and Non-Smokers in the RPE gDNA cohort. Mean and standard deviation (SD), median, minimum (min) and maximum (max) values were used to describe the distributions of age. *From a non-parametric Mann-Whitney U test of means.

Clock	DF	Sum Sq	Mean Sq	F- value	Pr(>F)	FDR-adjusted p-Value	Significant (Yes/No)
Horvath	3	418.1703215	139.3901072	2.793016	0.060255	0.06025537	No
Hannum	3	348.803089	116.2676963	4.20822	0.014904	0.02235675	Yes
Skin & Blood	3	298.1122679	99.37075598	4.898102	0.007879	0.02235675	Yes

Table S5. One-way ANOVA results for the Horvath multi-tissue, Hannum and Skin & Blood epigenetic clocks using whole-blood derived gDNA from stratified groups within Southampton Cohort.

One-way ANOVA's were performed for the Horvath multi-tissue, Hannum and Skin &Blood epigenetic clocks. Benjamini- Hochberg FDR adjusted to account for multiple testing was applied to the F-Test values for each ANOVA (3xF-test values corrected for multiple testing. The Hannum and Skin & Blood epigenetic clocks displayed significant FDR-adjusted p-values and were selected for the TukeyHSD post-hoc test (Additional File 2, Table S6) to assess differences between groups.

Epigenetic Clock	Groups Tested	Significant (Yes/No)	Tukey's Multiple Comparisons test Adjusted *p*-Value
Hannum Clock	Normal Non-Smoker vs. AMD Non-Smoker	No	0.9769
	Normal Non-Smoker vs. Normal Smoker	No	0.0641
	Normal Non-Smoker vs. AMD Smoker	No	0.2401
	AMD Non-Smoker vs. Normal Smoker	Yes	0.0334
	AMD Non-Smoker vs. AMD Smoker	No	0.1325
	Normal Smoker vs. AMD Smoker	No	0.8811
Skin & Blood Clock	Normal Non-Smoker vs. AMD Non-Smoker	No	0.3915
	Normal Non-Smoker vs. Normal Smoker	No	0.2302
	Normal Non-Smoker vs. AMD Smoker	No	0.6037
	AMD Non-Smoker vs. Normal Smoker	Yes	0.0064
	AMD Non-Smoker vs. AMD Smoker	Yes	0.0335
	Normal Smoker vs. AMD Smoker	No	0.8678

Table S6. EAA comparison from DNAm age estimation analysis from the Horvath multi-tissue, Hannum and Skin & Blood epigenetic clocks using whole-blood derived gDNA from stratified groups within Southampton Cohort.

Samples from the Southampton Cohort were stratified to compare the following groups: Normal Non-Smoker (n=6), AMD Non-Smoker (n=5), Normal Smoker (n=10) and AMD Smoker (n=9). Individual p-values for each comparison were derived using an Ordinary One-way ANOVA with the TukeyHSD post-hoc test (*$p \leq 0.05$;***$p \leq 0.001$).

Tissue	Control Sample Number (n)	AMD Sample Number (n)	Method	Differential expression of RPTOR (Yes/No)	Log Fold Change (if applicable)	*p*-value	Reference
RPE/ Choroid	31	37	Microarray	No	N/A	N/A	[57]
RPE/ Choroid	9	9	Microarray	No	N/A	N/A	[58]
RPE/ Choroid	8	8	Bulk RNA-Seq	Yes	+1.57477103986035	0.020769021	[59]
RPE/ Choroid	2	1	Single cell RNA-seq	Yes	-0.016671905	0.301322297	[60]
RPE/ Choroid	106	23	Bulk RNA-seq	No	N/A	N/A	[61]

Table S7. Differential expression of RPTOR from published transcriptomes investigating gene expression changes in AMD RPE.

Additional references:

[57] Newman AM et al. Systems-level analysis of age-related macular degeneration reveals global biomarkers and phenotype-specific functional networks. Genome Med. 2012. 4(2):16.

[58] Whitmore SS et al. Altered gene expression in dry age-related macular degeneration suggests early loss of choroidal endothelial cells. Mol Vis. 2013. 19:2274–97.

[59] Kim EJ et al. Complete transcriptome profiling of normal and age-related macular degeneration eye tissues reveals dysregulation of anti-sense transcription. Sci Rep. 2018. 8(1):3040.

[60] Voigt AP. et al. Single-cell transcriptomics of the human retinal pigment epithelium and choroid in health and macular degeneration. Proc. Natl Acad. Sci. 2019. 116:24100–24107.

[61] Orcozo LD et al. Integration of eQTL and a Single-Cell Atlas in the Human Eye Identifies Causal Genes for Age-Related Macular Degeneration. Cell Rep. 2020. 30(4):1246-1259.e6.

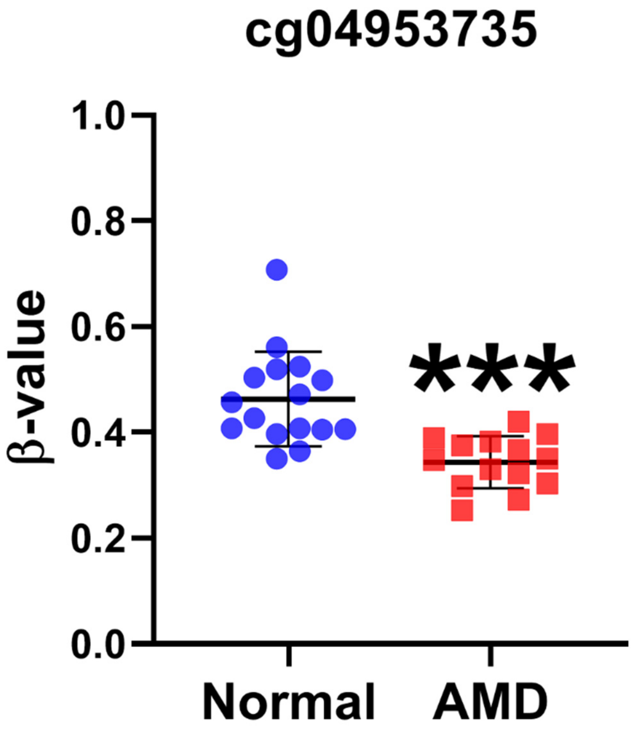

Figure S1. cg04953735 (RPTOR) displays a significant decrease in DNA methylation in human donor whole blood of AMD patients.

Methylation *β* value scatter plot for AMD (n=14) compared to Normal (n=16) human donor whole blood gDNA for cg04953735 (*RPTOR*). DNA methylation *β* values were significantly reduced in AMD compared to Normal human donor whole blood gDNA samples. (****p*=0.0001) All statistical analysis was performed using the parametric unpaired t-test.
